# Trajectories of psychological distress and spinal pain in manual therapists during the COVID-19 pandemic in Sweden

**DOI:** 10.1038/s41598-026-42074-1

**Published:** 2026-04-22

**Authors:** Nathan Weiss, Iben Axén, Trynke Hoekstra, Eva Skillgate

**Affiliations:** 1 Department of Health Promotion Science, Musculoskeletal and Sports Injury Epidemiology Center, Sophiahemmet University, 114 86 Stockholm, Sweden; 2 Unit of Intervention and Implementation Research for Worker Health, Institute of Environmental Medicine, Karolinska Institutet, 171 77 Stockholm, Sweden; 3 The Research Network Et Liv I Bevegelse (ELIB), Oslo, Norway; 4 Department of Health Sciences and Amsterdam Public Health Research Institute, Vrije Universiteit Amsterdam, Boelelaan 1105, 1081 HV Amsterdam, The Netherlands; 5 Naprapathögskolan-Scandinavian College of Naprapathic Manual Medicine, 114 19 Stockholm, Sweden

**Keywords:** Anxiety, Back pain, Chiropractor, Depression, Naprapath, Stress, Public health, Epidemiology, Pain, Anxiety, Depression

## Abstract

The aim of this study was to assess the one-year trajectories of psychological distress and of spinal pain intensity in manual therapists during the COVID-19 pandemic in Sweden, and to assess characteristics’ association with trajectory membership. In this cohort study, 816 clinically active licensed manual therapists answered four web-based surveys during a year of the COVID-19 pandemic in Sweden, measuring psychological distress and spinal pain intensity. Trajectory modelling was conducted to cluster individual trajectories over time. Association between baseline characteristics and cluster membership of psychological distress, and spinal pain intensity trajectories were analyzed using multinomial logistic regression models, adjusted for posterior probability, age, and sex. Five clusters of psychological distress and five of spinal pain intensity were identified. A majority of participants’ psychological distress (89%), and spinal pain intensity (75%) were clustered into low/mild levels. One cluster of increasing psychological distress (2%) were identified. Two fluctuating clusters of moderate/mild (14%), as well as one cluster of stable moderate (11%) spinal pain intensity were identified. Impaired sleep, and not meeting physical activity recommendations were associated with worse trajectories of spinal pain. In conclusion, most manual therapists had favorable trajectories of psychological distress and spinal pain intensity during a year of the COVID-19 pandemic in Sweden.

**Supplementary Information:**

The online version contains supplementary material available at 10.1038/s41598-026-42074-1.

## Introduction

The COVID-19 pandemic had an unprecedented global impact, including strain on healthcare systems, economic disruptions, and profound changes in the daily lives of people^1^. On May 4th, 2023, more than three years after the initial declaration by WHO, the end of the pandemic as a public health emergency was declared, with a total of 766 million confirmed cases worldwide, including 6.9 million reported deaths^2^.

Throughout the COVID-19 pandemic, governments and health authorities in different countries employed diverse strategies to mitigate the spread of SARS-CoV-2, while balancing the socioeconomic impact of restrictions^1^. Early concerns were raised regarding the mental and physical health impact in the general population of contracting SARS-CoV-2. Further, there were also concerns regarding the indirect effects of public health measures, such as social distancing, and stressors related to the pandemic at large^3^.

Healthcare workers faced a substantial burden during the pandemic as a result of the rapid viral transmission and conditions associated with COVID-19, and of the uncertainty and sudden changes in clinical routines^4^. Manual therapists treat, diagnose, and rehabilitate patients with musculoskeletal pain primarily using their hands, which necessitates close physical contact with the patient. Thus, these therapists had a risk of contracting and infecting the patient with SARS-CoV-2 in their clinical practice^5,6^. In Sweden, chiropractors and naprapaths are the two largest manual therapy professions licensed by the National Board of Health and Welfare^7,8^. Reports indicate that healthcare workers in Sweden had a higher incidence of SARS-CoV-2 infection than the general population during the early phases of the pandemic^9^. Further, occupational stress related to personal and colleagues’ safety has been reported among chiropractors amidst the pandemic^10^.

In cross-sectional data during the early phases of the pandemic among chiropractors, 50% and 30% reported moderate and high levels of psychological distress, respectively^10^. However, cross-sectional baseline data based on our cohort of manual therapists during the second wave of the COVID-19 pandemic in Sweden indicated low levels of psychological distress, and high prevalence of musculoskeletal pain, particularly back pain^11^, which corresponds to earlier studies reporting a high prevalence of musculoskeletal pain in manual therapists^12,13^.

As psychological distress and musculoskeletal pain typically show temporal inter- and intra-individual variability^14,15^, group-averaging models such as prevalence might mask important heterogeneity and unobserved subgroups within the population^16^. Identification of distinct subgroups of longitudinal psychological distress and musculoskeletal pain through trajectory modelling within a population could potentially have clinical implications. The identification of specific subgroups in a population that show poor health trajectories, and associated factors can inform the need of future interventions^17^. Notably, distinct trajectory subgroups of psychological distress and musculoskeletal pain, in particular low back pain, have been identified and show similarities across diverse populations^18,19^. In the context of the COVID-19 pandemic, subgroups of psychological distress and musculoskeletal pain trajectories with no/minimal, low, moderate, and increasing/elevated levels of psychological distress, distinct from the group mean have been identified^20–22^. Furthermore, lifestyle factors such as physical activity^23,24^ and sleep^25,26^, social support^26^, and the ability to cope with stressors ^27^ have previously been associated with psychological distress and back pain. However, the longitudinal trajectories of psychological distress and spinal pain in manual therapists during the COVID-19 pandemic is not known.

The aim of this study was to assess the one-year trajectories of psychological distress and of spinal pain intensity in manual therapists during the COVID-19 pandemic in Sweden, and to assess characteristics’ associations with trajectory membership.

## Material and methods

This study has a cohort design and is based on the Corona and Manual Professions (CAMP) study, ClinicalTrials register identifier: NCT04834583. The study was conducted according to the guidelines of the Declaration of Helsinki and approved by the Swedish Ethical Review Authority (Dnr: 2020-03836). The reporting of this study is in accordance with STROBE guidelines^28^. All participants provided electronic informed consent prior to inclusion in the study. Clinically active chiropractors and naprapaths, licensed by the National Board of Health and Welfare in Sweden, or those undergoing licensing practice after finishing their undergraduate training, were included. Extensive details regarding the recruitment, data collection procedures, and methods of the CAMP study have been published previously^11^ and are hence described briefly below.

Participants answered a web-based baseline survey in November 2020, during the second wave of the pandemic in Sweden, and were followed for one year (at 3-, 6-, and 12- months of follow-up to November 2021), to measure profession-related factors, work environment, health-related factors, lifestyle factors, psychological factors, and business’ economy.

### Outcomes

Symptoms of psychological distress were measured with the Depression Anxiety Stress scale-21 (DASS-21)^29^. The DASS-21 consists of 21 statements whereupon respondents rated how much a particular statement applied to them over the past 7-days, on a 4-point Likert scale, ranging from 0 “did not apply to me at all” to 3 “applied to me very much, or most of the time” (with each score multiplied by 2); resulting in a total score between 0 and 126^30,31^. A continuous scale was used in the analyses (the total sum of each participant’s DASS-21 scores), which previously has been shown to have construct validity as a unidimensional measure of psychological distress^30,32,33^.

Spinal pain intensity was measured with the Nordic Musculoskeletal Questionnaire. Respondents were asked whether they had experienced any pain the preceding three-month period, in the neck, jaw, shoulders, upper back, elbows, wrists/hands, low back, hips/thighs, knees, ankles/feet, and other bodily areas (yes/no), at each time-point. Respondents reporting pain (yes) were asked to rate their average pain intensity on a Numeric Rating Scale (NRS) ranging from 0 (no pain) to 10 (worst imaginable pain) during the preceding three-month period^34^, for each location separately. The mean pain intensity of the three most prevalent pain locations reported at baseline^11^, low back, upper back, and neck, was used as a compound measure: ‘spinal pain intensity’.

### Potentially associated factors

Factors potentially associated with the outcomes, psychological distress, and spinal pain intensity were prespecified a priori based on published literature and clinical reasoning within the research group, aiming to capture modifiable lifestyle factors, psychological resources, and relevant background characteristics. Variable categorization and planned analyses were prespecified before model fitting.

#### Maladaptive coping

To investigate participants’ strategies to cope with different stressors, a Swedish version of the Brief COPE questionnaire was used^35^. The questionnaire consists of 28 statements divided into 14 coping strategies. Respondents rated each statement on a 4-point Likert scale, ranging from 1 “I haven’t been doing this at all” to 4 “I have been doing this a lot”^36^. The 14 strategies are further divided into adaptive and maladaptive coping, with venting, denial, substance use, behavioral disengagement, self-distraction, and self-blame (items 1, 3, 4, 6, 8, 9, 11, 13, 16, 19, 21, 26) classified as maladaptive with a continuous total score ranging from 12 to 48^37,38^.

#### Not meeting physical activity recommendations

Physical activity (PA) was assessed with two questions adopted form the Swedish national public health survey (NBHWA-PA questions)^39,40^. Participants were asked how many minutes they committed to physical exercise in a normal week (e.g., running, fitness training, ball sports), and everyday PA (e.g., walking, cycling, gardening) with the following answer alternatives: “0 min/no time”, “1–30 min”, “30–60 min”, “60–90 min”, “90–120 min”, and “More than 120 min”. The categorical response alternatives were converted using the middle value in each category, e.g., 30–60 min were converted to 45 min. Further, each participant’s ‘total physical activity’ per week was calculated by summarizing their physical exercise multiplied with 2 (to account for the higher intensity of physical activity), with their minutes of everyday PA,^41^. Participants reporting < 300 min of total physical activity/week were classified as ‘not meeting physical activity recommendations’^42^.

#### Impaired sleep

To assess impaired sleep, the following questions were used: “Do you have difficulties falling asleep?”, “Do you wake up several times during the night and have difficulties falling asleep again?”, and “Do you feel very tired during worktime/daily activities?”. The answer alternatives were: “never”, “rarely/a few times per year”, “a few times per month”, “several times a week”, “always/every day”. The first two questions were adopted from the Karolinska Sleep Questionnaire^43^, and the last from the unwinding and recovery questions by Aronsson et al.^44^ Impaired sleep was defined as having difficulty initiating sleep and/or difficulty maintaining sleep accompanied by daytime consequences “several times a week” or “always/every day”^45^.

#### Business owner

At baseline, participants were asked “do you have your own business within chiropractic/naprapathy?”, those answering yes were considered business owners.

### Statistical analysis

Firstly, to inspect for inter- and intra-individual variability in the sample, the mean psychological distress and spinal pain intensity scores were plotted at each time-point for the whole sample. Group-level and individual-level psychological distress and spinal pain intensity were plotted with spaghetti plots to visually inspect the data and compared with the mean of the sample. Participants with internal missing answers of the outcome variables at all four time-points were excluded from respective analysis. No imputation methods were used for missing data. Secondly, Group-Based Trajectory Modelling (GBTM)^46^ and Growth Mixture Modelling (GMM)^47^ were conducted to identify distinct clusters of individual trajectories over the one-year study period for psychological distress, and spinal pain separately. Statistical analyses were conducted in R version 4.1.4^48^, GBTM and GMM were carried out with LCMM package with the ‘hlme’ and ‘lcmm’ functions^49^.

GBTM and GMM enable the identification of subgroups comprising individuals with similar trajectories. While GBTM assumes homogeneity within each subgroup, GMM accounts for individual variability within subgroups by estimating the within-class variance of growth factors, at intercept and slope^16^. The reporting and steps of the GBTM and GMM was done in accordance with the GRoLTS-Checklist^50^. Sample-size adjusted Bayesian Information Criteria (SA BIC) were used as goodness of fit (with lower values indicate a better fit), and average highest posterior probabilities (minimally 0.8), entropy near 1.0, cluster size were used for model selection, together with clinical and practical interpretability^16^. Based on previous research, four clusters were hypothesized a priori for psychological distress (resilient, moderate stable, recovery, and chronic), and musculoskeletal pain (minor pain/recovery, moderate pain, fluctuating pain, severe pain), separately^18,19^. The analyses followed a common stepwise procedure, starting with simple linear models with one cluster and subsequently increasing the number of clusters to six (two more than hypothesized a priori). Subsequently, the models were expanded with quadratic shape, and if the quadratic shape was statistically significant, and fit indices improved compared linear models, the process was thereafter repeated with cubic shape and thereafter equidistant splines, adding random effects at intercept, and then intercept and slope at each shape before proceeding to more complex models^16,51^. Each model was run for 200 iterations, randomly generating initial values from each model’s one cluster solution using gridsearch(), to reduce the odds of convergence towards local maxima^52^. Participants were classified into their best-fitting cluster based on the highest posterior cluster-membership probability from the trajectory model.

Thirdly, after choosing the final models, univariable and multivariable multinomial logistic regression models were built to assess the association between the independent variables ‘maladaptive coping’, ‘not meeting physical activity recommendations’, and ‘impaired sleep’ and the dependent variable cluster membership, with the cluster of lowest distress/spinal pain as the reference category. The multivariable models were adjusted for maximum posterior probability, age (continuous), and sex (male/female), with separate models for each independent variable. As cluster membership assignment is treated as error-free, we included each participant’s maximum posterior probability as a covariate in the multinomial logistic regression models to adjust for classification certainty. All regression models were prespecified; no stepwise or p-value-driven variable selection was used. Results from the multinomial logistic regression models are presented as odds ratios (OR) with corresponding 95% confidence intervals (95% CI).

In the descriptive statistics used to present baseline characteristics of the study sample, stratified by cluster membership, means and standard deviations (SDs) were calculated for continuous variables, whereas frequencies and proportions were calculated for categorical variables. Plots of mean psychological distress/spinal pain intensity for each cluster were made using GGplot2 in R^53^.

## Results

**Fig. 1:**
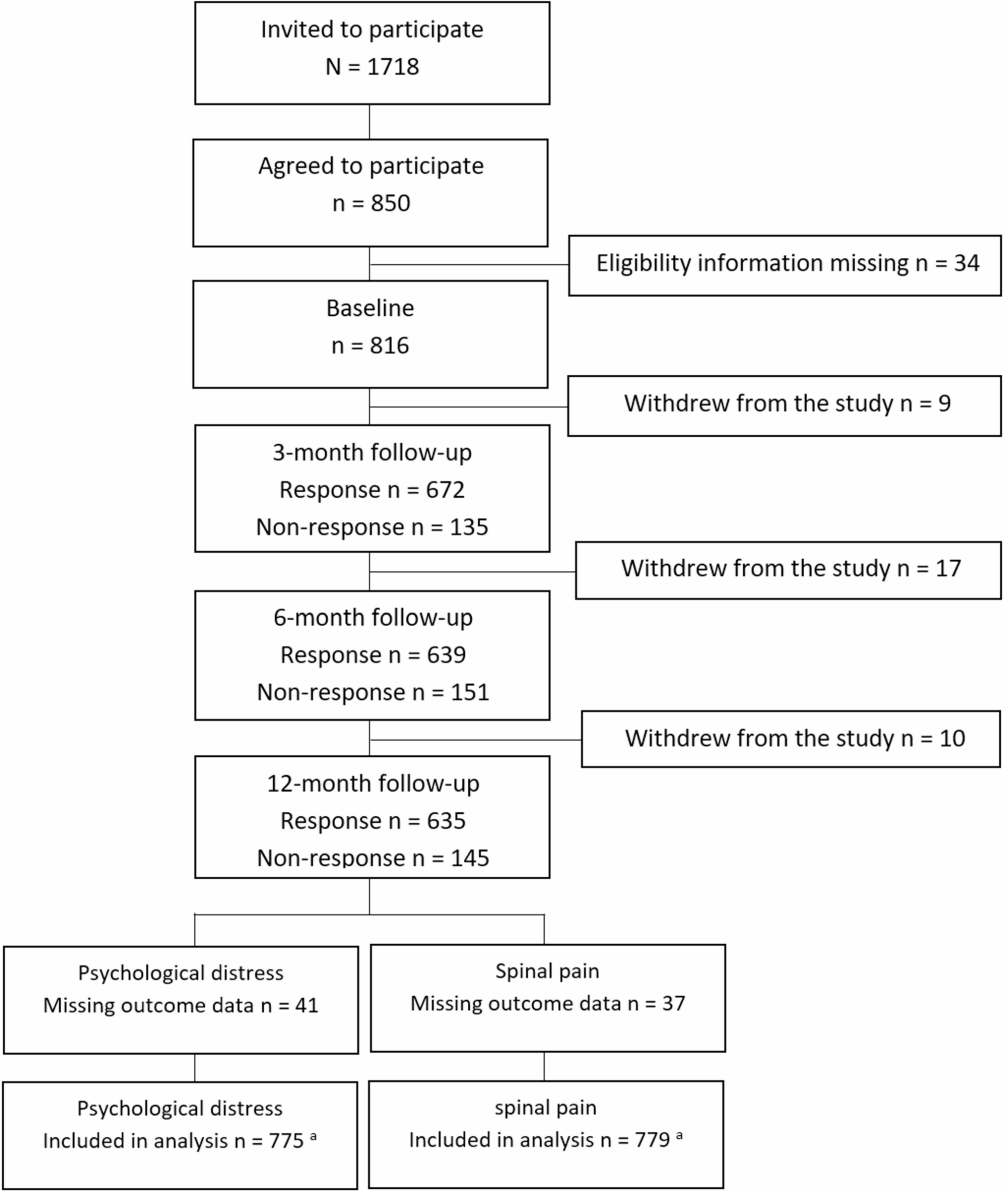
Study flow chart showing the number of participants invited, agreeing to participate, answering at each time-point, and in included in the analyses. ^a^ The number of participants with at least one time-point of valid outcome data were included in respective analysis.

In the CAMP cohort, a total of 1718 participants were invited, and 850 agreed to participate. Due to not providing information regarding eligibility criteria, 34 participants were excluded, resulting in a baseline sample of 816 clinically active manual therapists. Over the follow-up period of 12 months, a total of 36 participants dropped out of the study, and the response rate was around 80% at each follow-up. Due to internal missing outcome data at all four time-points, 41 participants for psychological distress, and 37 participants for spinal pain were excluded from the analyses. Consequently, the final study samples for psychological distress and spinal pain intensity were 775 and 779 participants with at least one time-point of outcome data, respectively (Fig. 1). The mean age of the sample was 43 (SD 11) years with an equal sex distribution (47% females).

### Trajectories

The total sample’s mean values of psychological distress and spinal pain intensity were stable over the 12-month follow-up period (see Supplementary Fig. S1). Plots of the participant’s individual trajectories of psychological distress (Supplementary Fig. S2), and spinal pain intensity (Supplementary Fig. S3), indicate the extent of the inter-individual variability in the sample.

GBTM with linear curves showed the best model fit for psychological distress, meaning participants in each identified cluster followed a similar trajectory, had and little inter-individual variability. Cubic shaped GMM with random intercept and slope showed the best model fit for spinal pain intensity, meaning participants’ growth parameters was non-linear during the time-period, and had inter-individual variability within each cluster. Table 1 presents the SA BIC, entropy, posterior probabilities, and cluster sizes across linear GBTMs for psychological distress and cubic shaped GMMs with random intercept and slope for spinal pain intensity ranging from 1–6 clusters. Models with splines and equidistant nodes (n = 4) resulted in convergence errors for both psychological distress and spinal pain intensity.

**Table 1:** SA BIC, posterior probabilities, and proportions of psychological distress, and spinal pain intensity.

No. clusters	SA BIC	Entropy	Posterior probabilities	Participants (%)
*Psychological distress, linear, no random effects (GBTM)*				
1	21,768	1.0	1.0	100
2	20,590	0.91	0.94/0.83	17/83
3	20,210	0.87	0.96/0.88/0.94	67/27/6
4	20,011	0.87	0.95/0.87/0.96/0.90	2/26/62/10
5	19,964	0.88	0.87/0.95/0.89/0.93/0.93	26/61/9/2/2
6	19,926	0.85	0.93/0.77/0.95/0.87/0.95/0.81	2/20/61/9/2 /6
*Spinal pain intensity, third order polynomial (cubic), random intercept and slope (GMM)*				
1	10,187	1.0	1.0	100
2	10,075	0.76	0.94/0.88	79/21
3	10,032	0.77	0.82/0.92/0.82	6/76/18
4	10,049	0.66	0.85/0.82/NA/0.81	76/6/0/18
5	9927	0.77	0.77/0.77/0.93/0.91/0.81	10/22/4/53/11
6	9996	0.60	NA/0.80/0.68/0.74/0.80/0.69	0/8/55/15/4 /18

*SA BIC* sample-size adjusted Bayesian information criterion.

For psychological distress, choosing the final number of clusters was difficult, as although BIC-scores kept decreasing with increasing numbers of clusters, the sizes of the additional identified clusters were small and not clearly distinct from other clusters. Therefore, for psychological distress, we balanced the statistical model fit criteria with careful inspection of the identified clusters in comparison to competing models and chose the model with 5 clusters. For spinal pain intensity, choosing the final model was relatively straightforward with BIC-scores decreasing with increasing numbers of clusters up to 5 total clusters. The optimal model for spinal pain intensity was therefore the model with 5 trajectories as well.

**Fig. 2:**
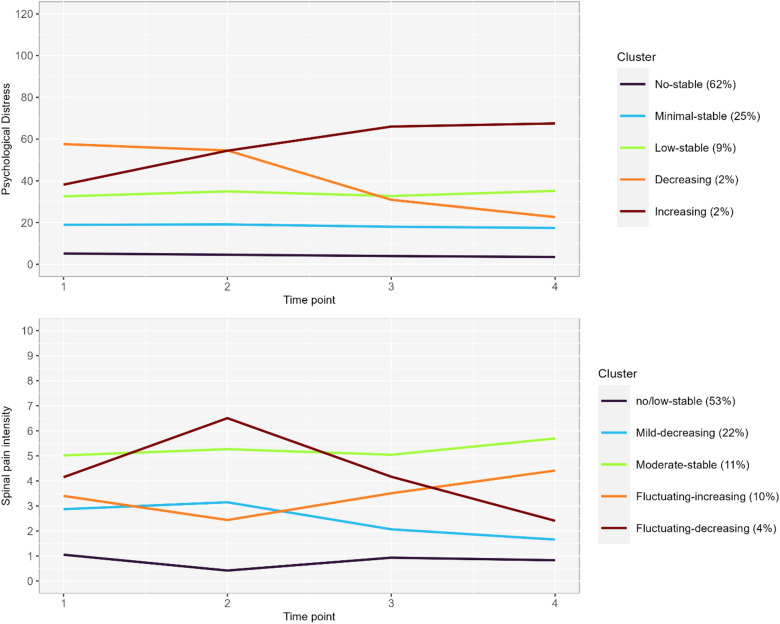
Mean psychological distress (top) and spinal pain intensity (bottom) at each time-point by cluster.

For both psychological distress and spinal pain intensity, posterior probabilities and entropy levels were generally above the 0.80 threshold for both optimal models, showing acceptable differentiation between clusters. All GBTMs and GMMs for psychological distress, and spinal pain intensity, respectively, are presented in Supplementary file, Tables S1–S6. Figure 2 presents the mean trajectories of psychological distress and spinal pain intensity over the follow-up period, for each cluster. Plots of competing models are presented in Supplementary file, Figs. S4–S13.

### Psychological distress

Most participants (62%) were assigned to cluster 1 (no-stable), characterized by a pattern of no to minimal levels of distress throughout the one-year period (Fig. 2). Cluster 2 (minimal-stable, 25%) and 3 (low stable, 9%) consisted of participants with varying degrees of constant low levels of psychological distress during follow-up. Cluster 4 (decreasing, 2%) initially had moderate levels of psychological distress with a minimal decline at three months of follow-up, followed by a gradual decrease, whereas cluster 5 (increasing, 2%) initially had mild values of psychological distress, followed by a gradual increase towards moderate values.

### Spinal pain intensity

Most participants (53%) were assigned to cluster 1 (no/low-stable) for spinal pain intensity, characterized by a pattern of no to low levels of pain throughout the one-year period (Fig. 2). Cluster 2 (mild-decreasing, 22%) had an intercept of mild pain intensity, with a slight increase at three-months of follow-up, followed by a gradual decrease over time within mild pain levels. Cluster 3 (moderate-stable, 11%) showed relatively constant moderate pain during the 12-month period. Furthermore, cluster 4 (fluctuating-increasing, 10%) consisted of participants with initial mild pain, and a slight decrease at three-months of follow-up, followed by a gradual increase in pain intensity reaching moderate levels at 12 months of follow-up. Cluster 5 (fluctuating-decreasing, 4%) initially had moderate pain intensity with a steep increase at three months, followed by a rapid recovery, and mild pain at the end of follow-up.

Baseline characteristics for the sample, stratified by cluster membership of psychological distress, and spinal pain intensity are presented in Tables 2 and 3, respectively.

**Table 2:** Baseline characteristics, stratified by psychological distress cluster membership.

	No-stable n = 478 (62%)	Minimal-stable n = 199 (25%)	Low-stable n = 69 (9%)	Decreasing n = 15 (2%)	Increasing n = 14 (2%)	Total N = 775 (100%)
Age, mean (SD)	45.4 (11.5)	42.6 (10.7)	41.2 (10.3)	42.7 (10.8)	37.9 (7.5)	44.1 (11.3)
*Sex, n (%)*						
Male	274 (57)	97 (49)	33 (48)	6 (40)	4 (29)	414 (53)
Female	204 (43)	102 (51)	36 (52)	9 (60)	10 (71)	361 (47)
Number of hours clinically active/week previous 3 months, mean (SD)	29.3 (11.3)	26.8 (11.0)	25.5 (12.2)	27.8 (12.8)	27.5 (10.0)	28.3 (11.4)
Business owner, n (%)*	343 (74)	146 (77)	46 (70)	14 (93)	8 (62)	557 (75)
Regular tobacco consumption, n (%)*						
Smoking	1 (0)	2 (1)	0 (0)	0 (0)	0 (0)	3 (0)
Snus	65 (14)	43 (22)	9 (13)	1 (7)	1 (8)	119 (16)
Number of days physically active/week, mean (SD)*	3.9 (1.6)	3.5 (1.6)	3.5 (1.5)	4.0 (2.0)	3.6 (1.9)	3.8 (1.6)
Not meeting physical activity recommendations, n (%)*^a^	199 (43)	104 (54)	37 (55)	9 (60)	10 (77)	260 (52)
Impaired sleep, n (%)*^b^	14 (3)	28 (15)	15 (22)	4 (27)	5 (38)	66 (9)
Maladaptive coping, mean (SD)*^c^	20.2 (4.1)	22.7 (3.6)	25.2 (3.9)	25.5 (2.6)	27.8 (5.4)	21.5 (4.4)

*Internal missing, maximum 8%. ^a^Less than 300 min of moderate or vigorous intensity physical activity/week. ^b^Difficulties falling asleep and waking up during the night in combination with daytime interference of activities. ^c^Sum score of Maladaptive coping strategies of Brief COPE, items: 1, 3, 4, 6, 8, 9, 11, 13, 16, 19, 21, 26.

**Table 3:** Baseline characteristics, stratified by spinal pain cluster membership.

	No/low-stable n = 413 (53%)	Mild-decreasing n = 174 (22%)	Moderate-stable n = 82 (11%)	Fluctuating-increasing n = 81 (10%)	Fluctuating-decreasing n = 29 (4%)	Total N = 779 (100%)
Age, mean (SD)	44.7 (11.8)	42.9 (11.2)	44.9 (10.1)	45.0 (10.3)	39.4 (8.3)	44.1 (11.3)
*Sex, n (%)*						
Male	243 (59)	95 (55)	31 (38)	38 (47)	10 (34)	417 (54)
Female	170 (41)	79 (45)	51 (62)	43 (53)	19 (66)	362 (46)
Number of hours clinically active/week previous 3 months, mean (SD)	28.9 (11.5)	28.1 (10.7)	25.4 (11.7)	29.6 (10.8)	25.0 (12.1)	28.3 (11.4)
Business owner, n (%)*	300 (76)	120 (73)	56 (31)	62 (78)	19 (70)	557 (75)
Regular tobacco consumption, n (%)*						
Smoking	2 (0)	1 (1)	0 (0)	0 (0)	0 (0)	3 (0)
Snus	70 (17)	25 (15)	9 (12)	10 (12)	5 (19)	119 (16)
Number of days physically active/week, mean (SD)*	4.0 (1.6)	3.6 (1.7)	3.6 (1.7)	3.2 (1.5)	3.5 (1.9)	3.8 (1.6)
Not meeting physical activity recommendations, n (%)*^a^	229 (57)	83 (50)	35 (45)	37 (46)	12 (44)	396 (52)
Impaired sleep, n (%)*^b^	21 (5)	15 (9)	18 (23)	9 (11)	3 (11)	66 (9)
Maladaptive coping, mean (SD)*^c^	20.9 (4.3)	22.0 (4.7)	22.9 (3.7)	22.1 (4.5)	22.3 (3.2)	21.5 (4.4)

*Internal missing, maximum 8%. ^a^ Less than 300 min of moderate or vigorous intensity physical activity/week. ^b^ Difficulties falling asleep and waking up during the night in combination with daytime interference of activities. ^c^ Sum score of Maladaptive coping strategies of Brief COPE, items: 1, 3, 4, 6, 8, 9, 11, 13, 16, 19, 21, 26.

### Associated factors

Results from the univariable and multivariable multinomial logistic regression models of baseline characteristics’ association with cluster membership of psychological distress, and spinal pain intensity are presented in Tables 4 and 5, respectively, in comparison with the cluster with lowest distress, and pain (cluster 1). Those reporting impaired sleep at baseline had an adjusted OR of 5.2-21.2 of belonging to a psychological distress cluster, and an adjusted OR of belonging to a spinal pain intensity cluster of 1.5-4.4. Furthermore, not meeting physical activity recommendations was associated with an adjusted OR of 1-8-4.9 of belonging to a psychological distress cluster, and an adjusted OR of 1.3-1.7 of belonging to a spinal pain cluster.

**Table 4:** Association between baseline characteristics and psychological distress cluster membership.

Variable	Crude OR (95% CI)					Adjusted OR (95% CI)^a^				
	No-stable n = 478 (62%)	Minimal-stable n = 199 (25%)	Low-stable n = 69 (9%)	Decreasing n = 15 (2%)	Increasing n = 14 (2%)	No-stable n = 478 (62%)	Minimal-stable n = 199 (25%)	Low-stable n = 69 (9%)	Decreasing n = 15 (2%)	Increasing n = 14 (2%)
Impaired sleep^b^	1.0 (ref)	5.5 (2.8–10.7)	9.4 (4.3–20.5)	11.9 (3.3–41.7)	20.3 (5.9–69.9)	1.0 (ref)	5.2 (2.6–10.3)	9.4 (4.2–20.9)	11.2 (3.2–40.4)	21.2 (5.9–76.2)
Not meeting physical activity recommendations^c^	1.0 (ref)	1.6 (1.1–2.2)	1.7 (1.0–2.8)	2.0 (0.7–5.8)	4.5 (1.2–16.6)	1.0 (ref)	1.8 (1.2–2.5)	1.8 (1.1–3.1)	2.1 (0.7–6.0)	4.9 (1.3–18.0)
Maladaptive coping^d,e^	1.0 (ref)	1.2 (1.1–1.2)	1.4 (1.3–1.5)	1.4 (1.2–1.6)	1.5 (1.3–1.7)	1.0 (ref)	1.2 (1.1–1.2)	1.4 (1.3–1.5)	1.4 (1.2–1.5)	1.5 (1.4–1.7)
Business owner^f^	1.0 (ref)	1.2 (0.8–1.8)	0.8 (0.5–1.4)	4.9 (0.6–37.7)	0.6 (0.2–1.7)	1.0 (ref)	1.7 (1.1–2.7)	1.2 (0.7–2.4)	7.9 (1.0–64.3)	1.3 (0.4–4.6)

*CI* confidence interval,* OR* odds ratio. ^a^ Adjusted for posterior probability, age and sex. ^b^ Difficulties falling asleep and waking up during the night in combination with daytime interference of activities. ^c^ Less than 300 min of moderate or vigorous physical activity/week. ^d^ Continuous measure of maladaptive coping items 1, 3, 4, 6, 8, 9, 11, 13, 16, 19, 21, 26 from Brief COPE, range 12–48. ^e^ One increase in exposure represents 1 point of total score on the Brief COPE maladaptive strategies. ^f^ Business owner (yes/no).

**Table 5:** Association between baseline characteristics and spinal pain intensity cluster membership.

Variable	Crude OR (95% CI)					Adjusted OR (95% CI)^a^				
	No/low-stable n = 413 (53%)	Mild-decreasing n = 174 (22%)	Moderate-stable n = 82 (11%)	Fluctuating-increasing n = 81 (10%)	Fluctuating-decreasing n = 29 (4%)	No/low-stable n = 413 (53%)	Mild-decreasing n = 174 (22%)	Moderate-stable n = 82 (11%)	Fluctuating-increasing n = 81 (10%)	Fluctuating-decreasing n = 29 (4%)
Impaired sleep^b^	1.0 (ref)	1.8 (0.9–3.6)	5.4 (2.7–10.6)	2.3 (1.0–5.2)	2.3 (0.6–8.1)	1.0 (ref)	1.5 (0.7–3.1)	4.4 (2.2–9.0)	1.8 (0.8–4.2)	2.0 (0.6–7.5)
Not meeting physical activity recommendations^c^	1.0 (ref)	1.3 (0.9–2.0)	1.3 (0.9–1.9)	1.6 (1.0–2.6)	1.6 (0.8–3.6)	1.0 (ref)	1.3 (0.9–2.0)	1.6 (1.0–2.6)	1.5 (0.9–2.5)	1.7 (0.8–3.7)
Maladaptive coping^d,e^	1.0 (ref)	1.1 (1.0–1.1)	1.1 (1.1–1.2)	1.1 (1.0–1.1)	1.1 (1.0–1.2)	1.0 (ref)	1.0 (1.0–1.1)	1.1 (1.0–1.1)	1.0 (1.0–1.1)	1.0 (0.9–1.2)
Business owner^f^	1.0 (ref)	0.9 (0.6–1.3)	0.8 (0.5–1.4)	1.2 (0.7–2.1)	0.8 (0.3–1.8)	1.0 (ref)	1.0 (0.6–1.6)	0.8 (0.4–1.5)	1.2 (0.6–2.2)	1.4 (0.6–3.7)

*CI* confidence interval, *OR* odds ratio. ^a^ Adjusted for posterior probability, age and sex. ^b^ Difficulties falling asleep and waking up during the night in combination with daytime interference of activities. ^c^ Less than 300 min of moderate or vigorous physical activity/week. ^d^ Continuous measure of maladaptive coping items 1, 3, 4, 6, 8, 9, 11, 13, 16, 19, 21, 26 from Brief COPE, range 12–48. ^e^ One increase in exposure represents 1 point of total score on the Brief COPE maladaptive strategies. ^f^ Business owner (yes/no).

## Discussion

In this cohort study of manual therapists during the COVID-19 pandemic in Sweden, five trajectories of psychological distress and five trajectories of spinal pain intensity were identified, respectively. These trajectories were, at large, relatively mild in terms of symptom severity. Only one trajectory of worsening psychological distress, comprising merely 14 participants (2% of the sample) was identified, demonstrating that the vast majority of manual therapists had low distress levels during the one-year follow-up. With regards to spinal pain intensity, two fluctuating clusters of moderate/mild, as well as one cluster of stable moderate spinal pain intensity were identified, comprising 192 individuals (25% of the sample). Reporting impaired sleep and not meeting physical activity recommendations at baseline were associated with worse spinal pain intensity trajectories during follow-up. Similarly, those reporting impaired sleep, not meeting physical activity recommendations, and higher scores of maladaptive coping strategies at baseline had a higher probability of belonging to a worse psychological distress trajectory during follow-up.

### Trajectories of psychological distress

Individual mental responses after macro-stressors (such as the COVID-19 pandemic) typically follow common characteristics, with four dominant trajectories reported: a resilient trajectory, a recovery trajectory, a chronic trajectory, a delayed trajectory (slow increase), and sometimes a moderate/mild stable trajectory^54^. Numerous studies conducted during the COVID-19 pandemic on distress in different populations have identified the aforementioned clusters^19^, which corresponds to the findings from our study, where we identified one resilient trajectory, two mild distress trajectories, one delayed increase trajectory, and one recovery trajectory, with similar proportions compared to other studies^19^. However, we did not identify any trajectory of chronically elevated distress in our sample, which could be because our sample were resilient and maintained healthy lifestyle behaviors throughout the pandemic, and generally had low symptoms of distress as presented in cross-sectional data based on our cohort^11^.

### Trajectories of spinal pain intensity

Studies examining musculoskeletal pain trajectories during the COVID-19 pandemic are scarce, and have been conducted in other settings, populations, and have used other means of classifying pain, which limits interstudy comparison. Nonetheless, our findings are consistent with much of the body of evidence of back pain trajectories, with most studies reporting cluster solutions representing no/occasional pain, mild stable pain, fluctuating patterns, persistent moderate/severe pain, and improving/recovering pain trajectories^18,55,56^.

### Associated factors

Participants reporting impaired sleep at baseline were more likely to have an unfavorable trajectory of psychological distress, and spinal pain intensity during follow-up, compared to participants without impaired sleep. Poor sleep quality has previously been reported as a risk factor for back pain and as a prognostic factor for future pain intensity and recovery^57,58^. Furthermore, sleep disturbances have been associated with developing pain and chronic pain trajectories in a 13-year prospective study^23^. The relationship between sleep quality and psychological distress has been studied extensively^59^, and sleep quality seem to predict future psychological distress^60^.

Those reporting less than 300 min of physical activity/week at baseline were more likely to have a worse trajectory of psychological distress, and spinal pain intensity. Similarly, engagement in moderate to vigorous physical activity has previously been associated with a lower probability of belonging to trajectories of severe back pain and worse psychological distress^24,25^.

Lastly, participants with higher scores of maladaptive coping strategies had a higher probability of belonging to a worse cluster of psychological distress in our study. A previous study conducted in China during the early stages of the COVID-19 pandemic found that maladaptive coping predicted distress trajectories^61^.

However, there there could be a risk of of reverse association, for example, participants in worse clusters reported relatively high pain intensity at baseline, which might have impaired their sleep, and caused this association. The same reasoning may be relevant for the association between not meeting physical activity recommendations and a worse trajectory of spinal pain intensity over the one-year follow-up. These observed relationships could also be bi-directional, considering we did not choose to study only participants without pain or distress at baseline. Consequently, participants could therefore already be experiencing psychological distress, spinal pain, and the associated factors studied before being enrolled, which limit the ability to elucidate the direction of associations. Considering all data points were collected during the COVID-19 pandemic, and there was no comparison with pre-pandemic estimates or time-points in the study population, it is not possible to ascertain whether the trajectories were influenced by the COVID-19 pandemic or not.

### Methodological discussion

The CAMP-cohort study included a large proportion of all licensed manual therapist in Sweden (47%) which is important for the external validity of this study ^11^. The response rate around 80% at each follow-up further strengthens the internal validity of the study.

The DASS-21-instrument has previously demonstrated adequate psychometric properties^29^, and using a continuous scale (the total sum of each participant’s DASS-21 scores) to measure unidimensional psychological distress, has good construct validity^30,32,33^.Even though measuring pain intensity with an 11-point numerical rating scale is considered the best way to capture pain intensity in questionnaires, the definition of the outcome spinal pain intensity may be considered a limitation as it was constructed as each participant’s mean of low back, upper back, and neck pain intensity. Pain in these areas may be considered separate entities influenced differently by age, sex, workload and other conditions, although some evidence suggests that spinal pain is indeed a construct that changes very little through age ^62^.

The small proportion of participants being assigned to cluster 4, and 5 of psychological distress, which is reflected in the low precision in the multinomial regression models, and wide confidence intervals, can be considered a limitation of our study. These results should be interpreted with caution. Future studies with larger sample size are needed to explore factors associated with specific psychological distress and spinal pain trajectories in manual therapists. Further, due to limitations in the statistical software R, no fit statistics such as LoMendel-Rubin-likelihood ratio test or the bootstrap likelihood ratio were performed. Furthermore, cluster membership used as the dependent variable in the multinomial logistic regression models was based on each participant’s most likely assignment (maximum posterior probability) in the trajectory models. Although we adjusted the regression models for the maximum posterior probability to account for the classification certainty, this approach does not fully correct for outcome misclassification and may bias associations.

Lastly, the employed statistical analysis is data-driven, with the assumption that distinct subgroups exist in the data. The strength of this type of analysis is that previously unknown classes may be unveiled. In order to evaluate the value of these clusters, they need to be compared to those found in other populations, similar to and different from the one under investigation. Further, the chosen models (five cluster solutions of psychological distress, and spinal pain intensity) had clusters below the 5% recommended threshold, as well as clusters with posterior probability below the 0.80 threshold. However, after careful inspection and discussion within the research group, these relatively small clusters, especially for psychological distress (two clusters consisting of 2%) were kept, as they were deemed to provide valuable information on the clinical course, and in line with similar trajectories compared to other studies on psychological distress^19^.

### Implications

The results from our study highlight the heterogeneity and inter-individual variability in psychological distress and spinal pain over time, and factors potentially associated with distinct trajectories. Further, the knowledge of associated factors, such as impaired sleep, not meeting physical activity recommendations, and maladaptive coping strategies can potentially be used for early identification of high-risk groups. In the context of public crises such as the COVID-19 pandemic, this information could be of importance to policy makers and for public health measures in future similar contexts. However, future studies with larger sample sizes are required to assess these associations further, especially with regards to psychological distress.

## Conclusions

A majority of manual therapists had a favorable trajectory of psychological distress and spinal pain intensity during a year of the COVID-19 pandemic in Sweden. However, a quarter of participants had unfavorable trajectories of symptoms of spinal pain and reporting impaired sleep, not meeting physical activity recommendations, and maladaptive coping were associated with higher probability of worse spinal pain trajectories. However, no firm conclusions can be made regarding associated factors of psychological distress trajectories considering low statistical power.

## Supplementary Information

Below is the link to the electronic supplementary material.

Supplementary Material 1

## Data Availability

The datasets generated and/or analyzed during the current study are not publicly available due to ethical considerations and the general data protection regulation (GDPR) but are available from the corresponding author on reasonable request.
