# Supplementary material for: Trajectories of psychological distress and spinal pain in manual therapists during the COVID-19 pandemic in Sweden
Source: Sci Rep. 2026 Apr 22;16:13150. doi: 10.1038/s41598-026-42074-1 (PMC13102993; doi:10.1038/s41598-026-42074-1)
Supplement: Supplementary file 1 — Supplementary Material 1 [file 41598_2026_42074_MOESM1_ESM.pdf]

Supplemental file

Trajectories of psychological distress and spinal pain in manual therapists during the COVID-19 pandemic in Sweden: the CAMP cohort study

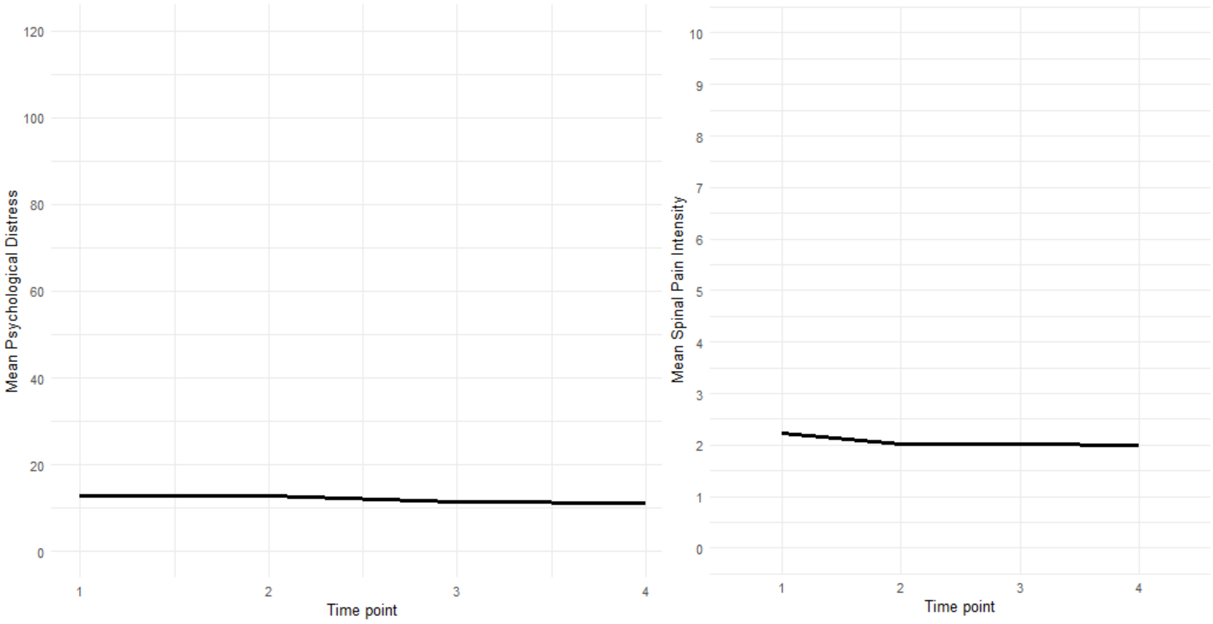

Figure S1. Mean values of psychological distress (left) and spinal pain (right).

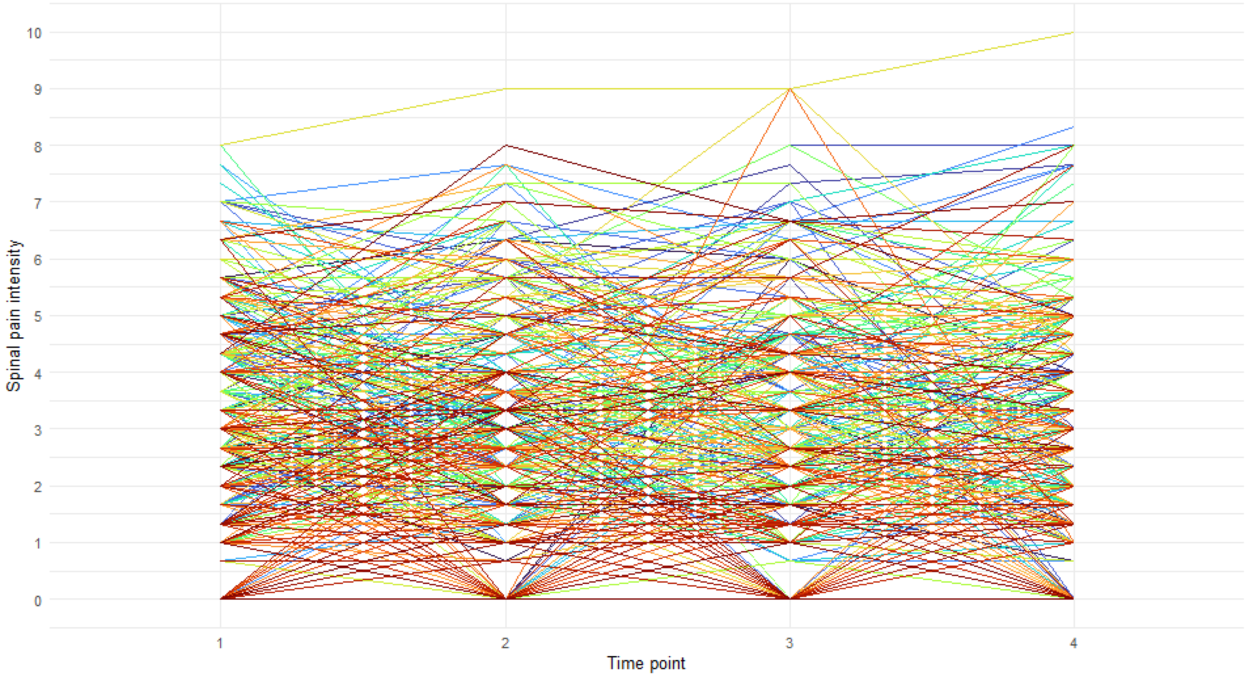

Figure S2. Spaghetti plot of spinal pain for the total sample.

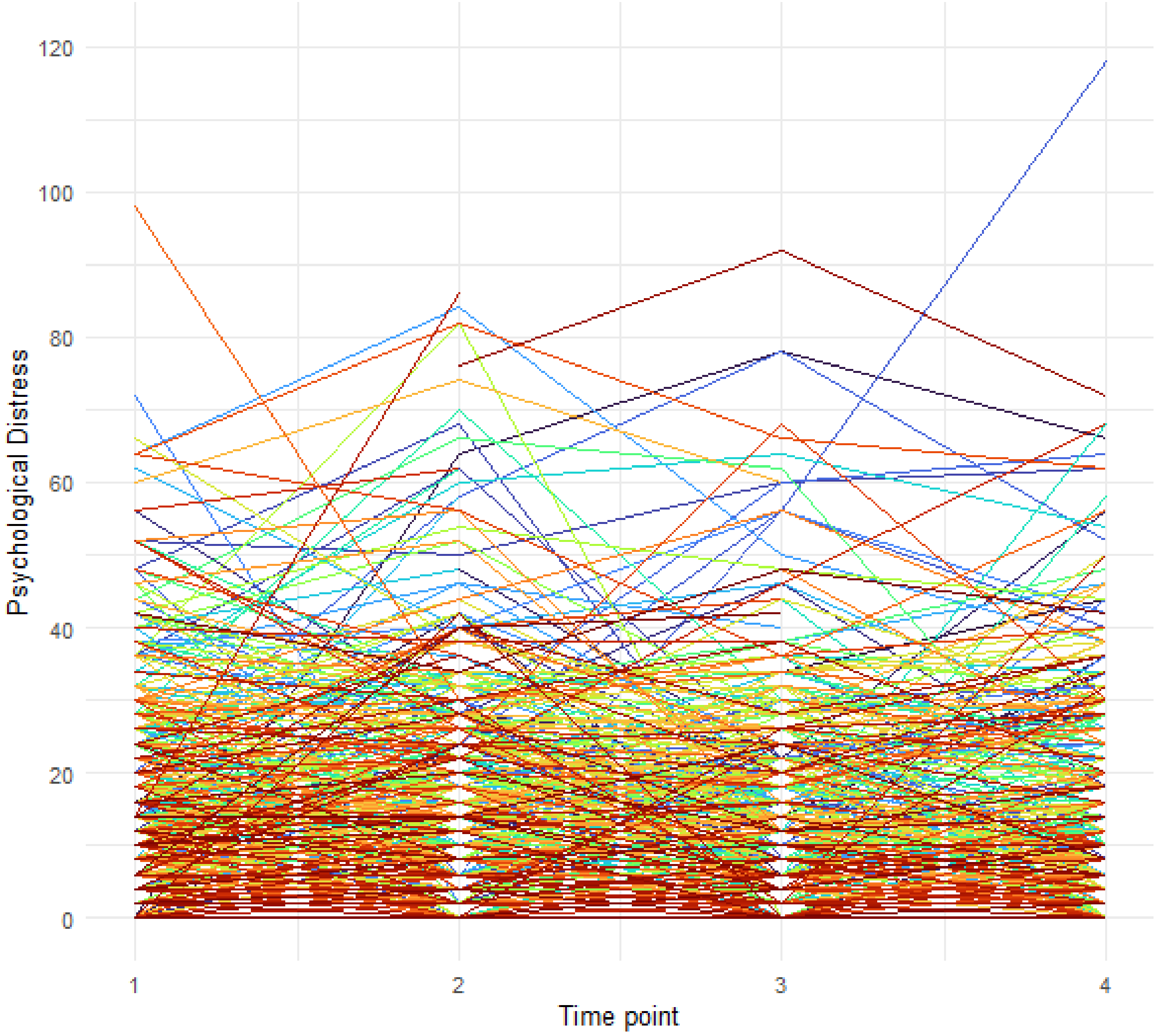

Figure S3. Spaghetti plot of psychological distress for the total sample.

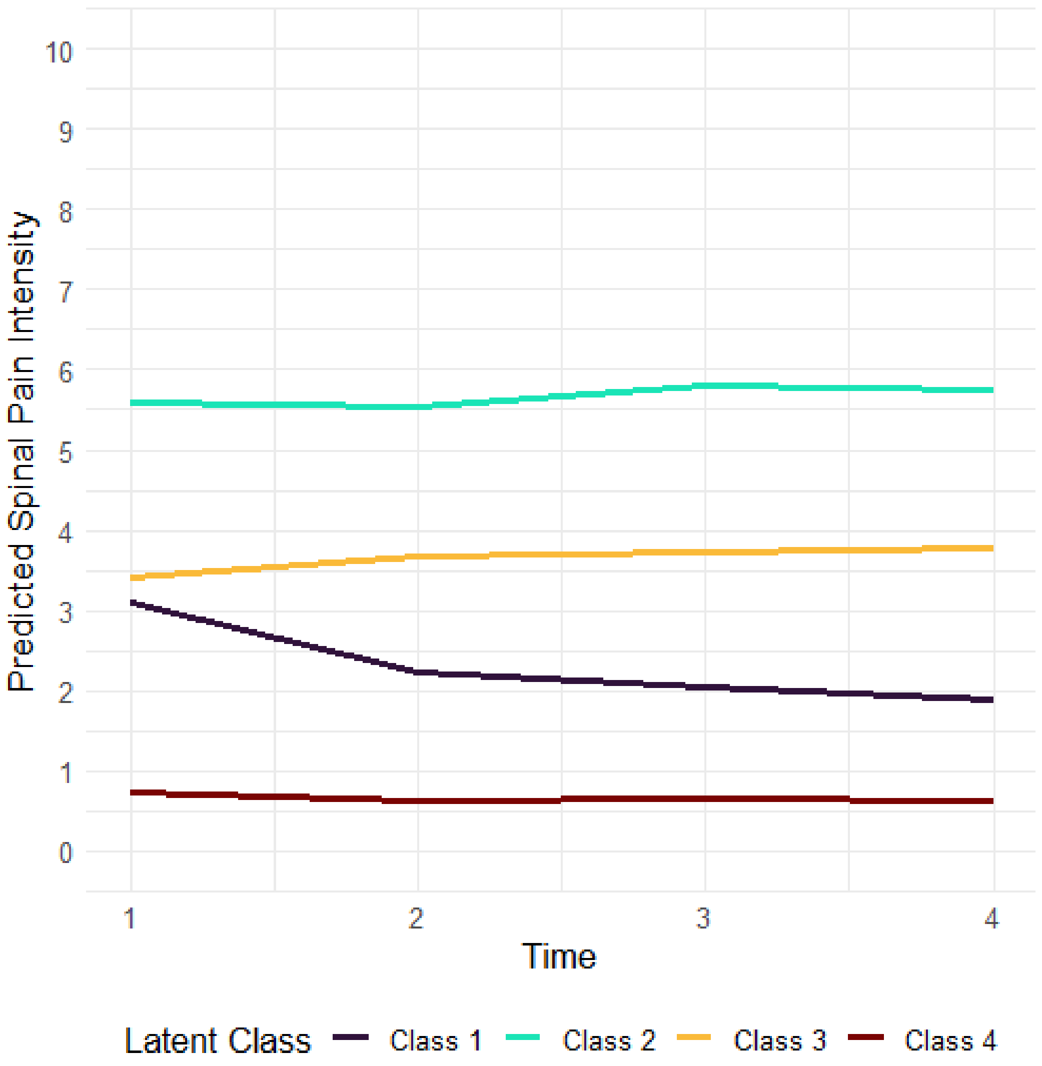

Figure S4. Predicted splines of spinal pain intensity GBTM, linear with no random effects consisting of 4 clusters.

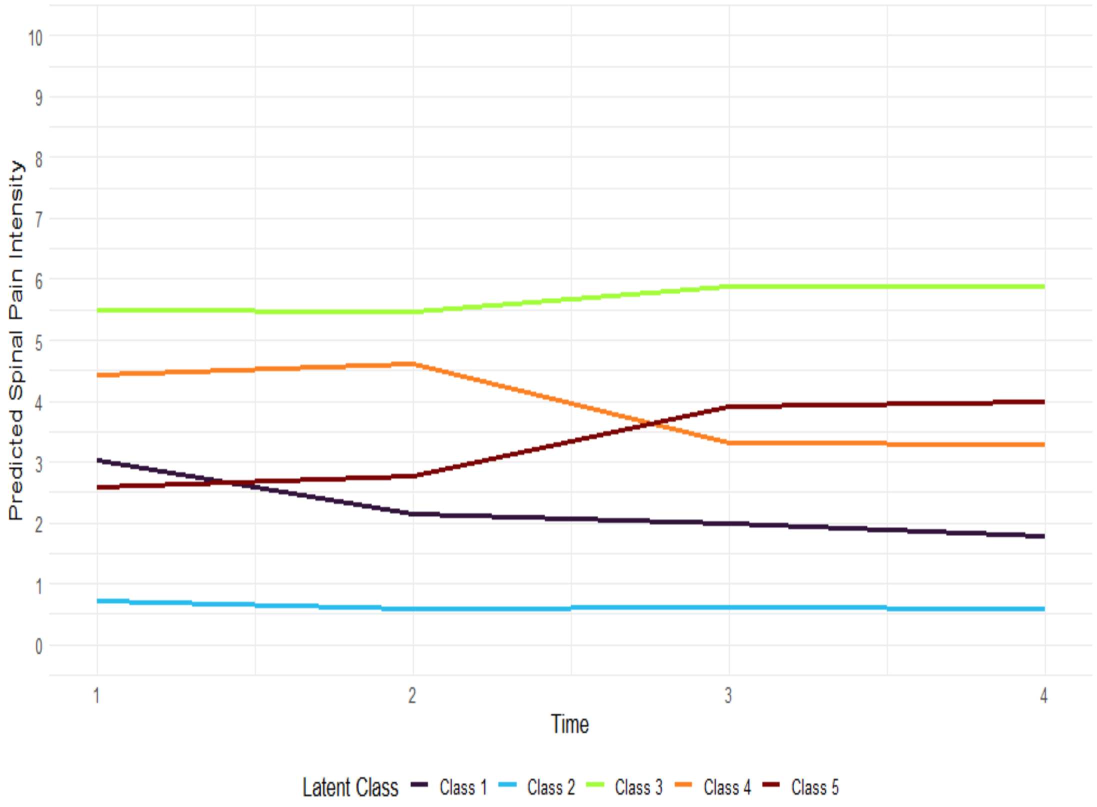

Figure S5 Predicted splines of spinal pain intensity GBTM, linear with no random effects consisting of 5 clusters.

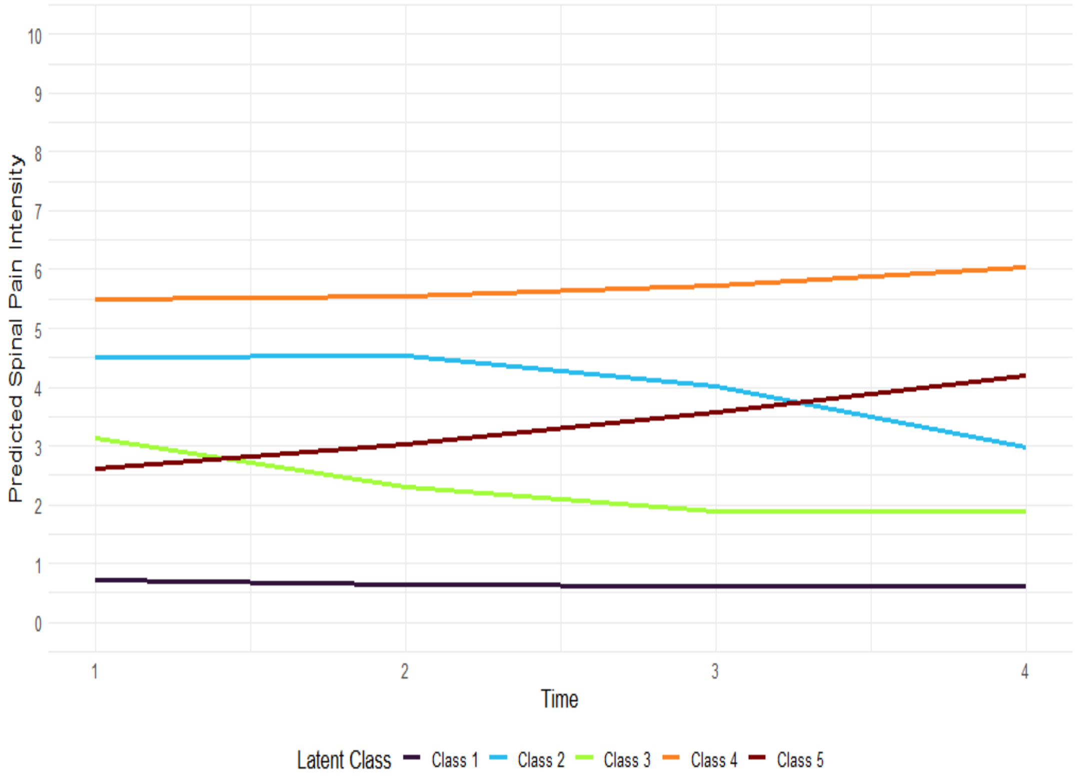

Figure S6. Predicted splines of spinal pain intensity GBTM, quadratic shape with no random effects consisting of 5 clusters.

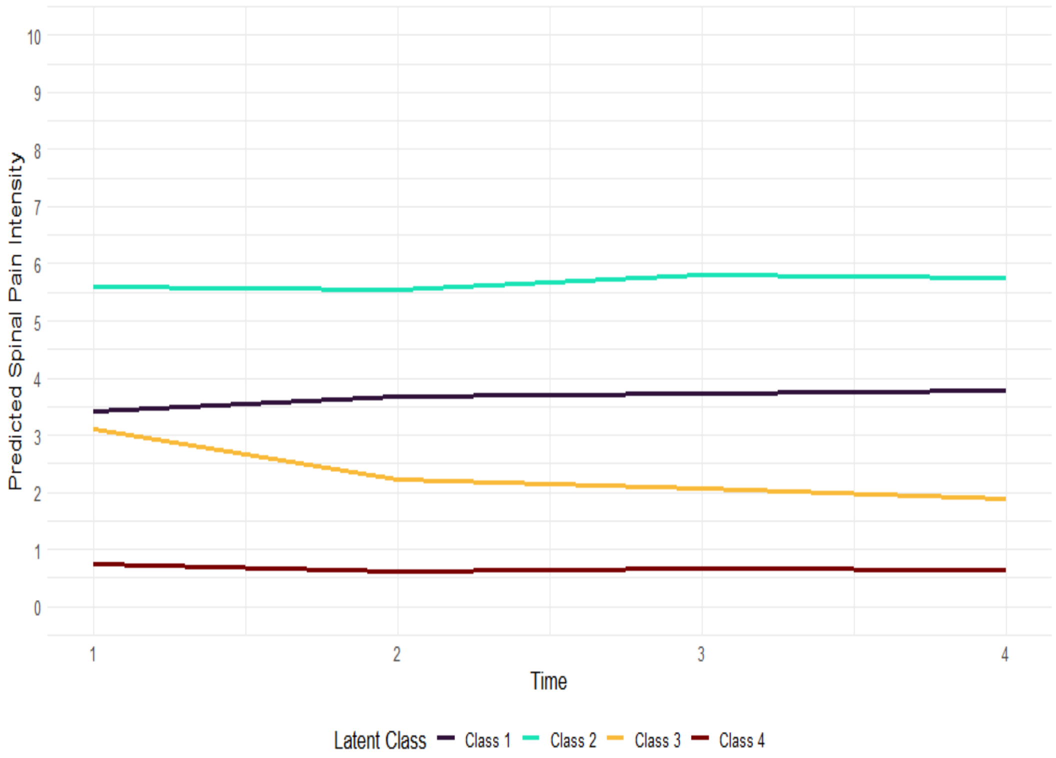

Figure S7. Predicted splines of spinal pain intensity GBTM, cubic shape with no random effects consisting of 4 clusters.

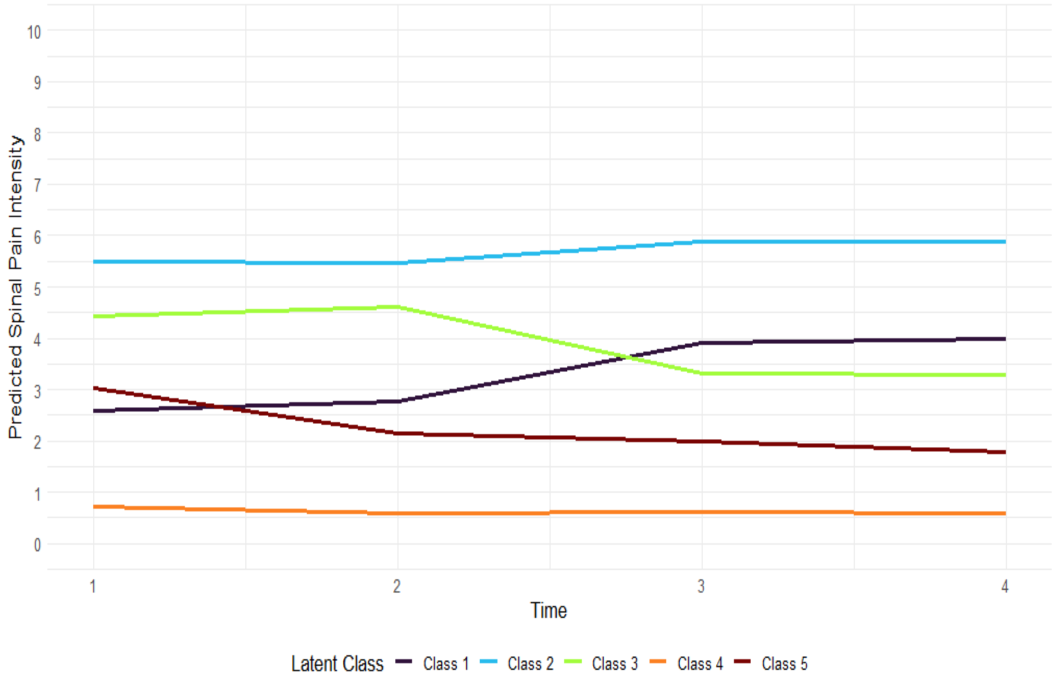

Figure S8. Predicted splines of spinal pain intensity GBTM, cubic shape with no random effects consisting of 5 clusters.

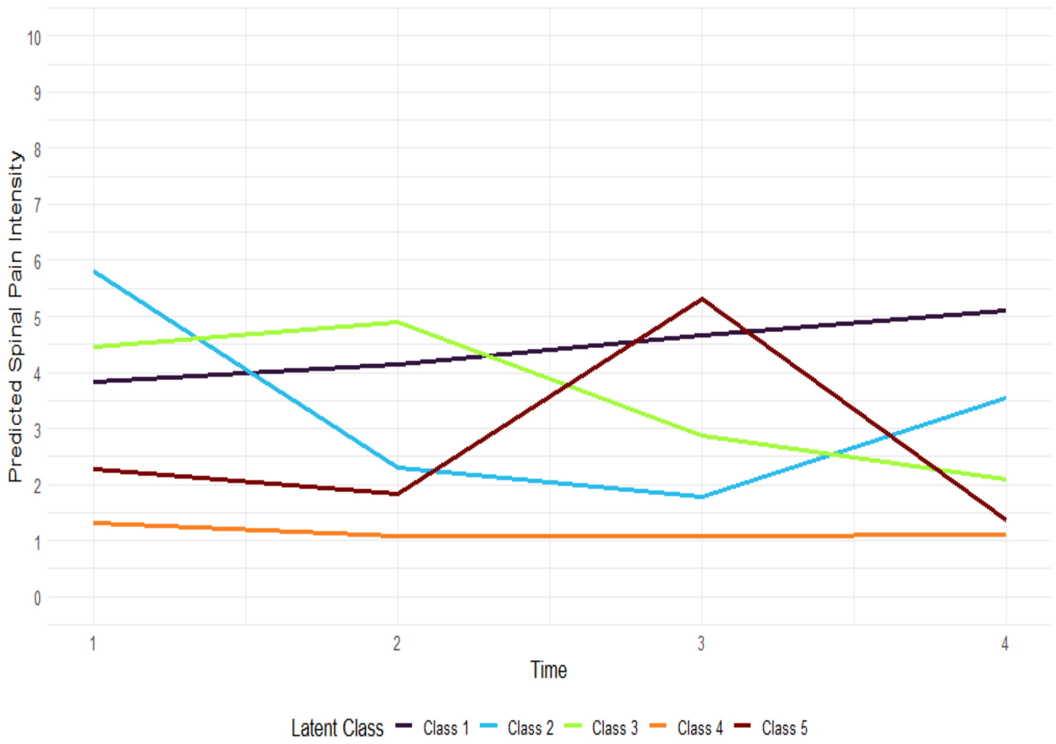

Figure S9. Predicted splines of spinal pain intensity GMM1, cubic shape with random intercept consisting of 5 clusters.

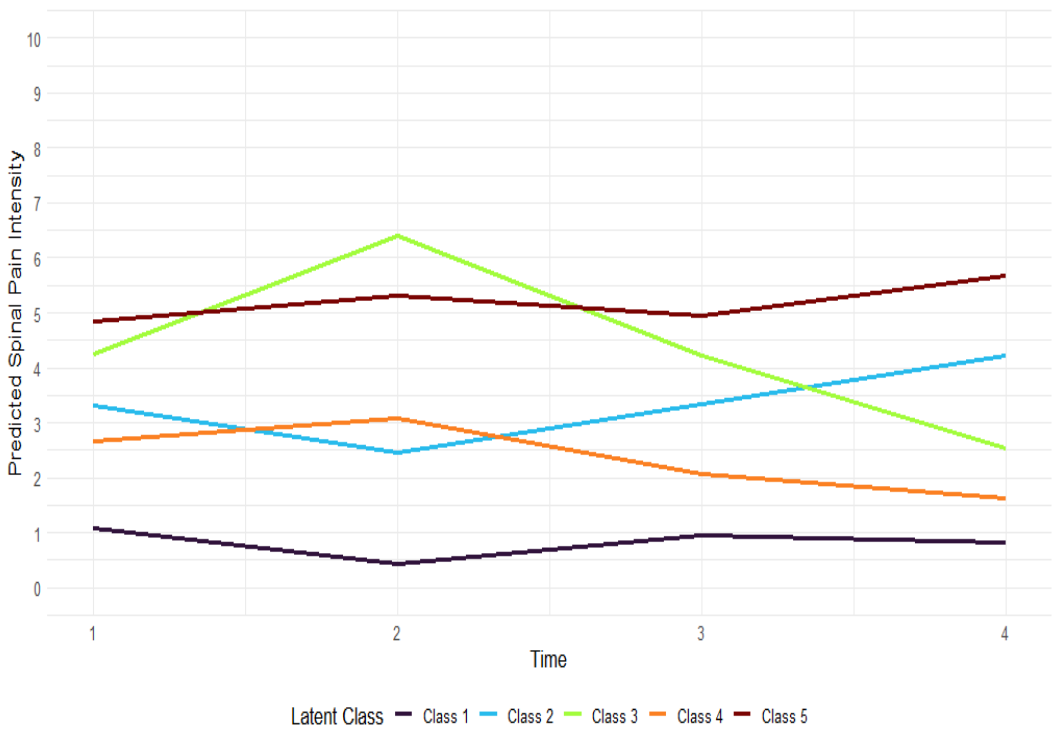

Figure S10. Predicted splines of spinal pain intensity GMM2, cubic shape with random intercept and slope consisting of 5 clusters.

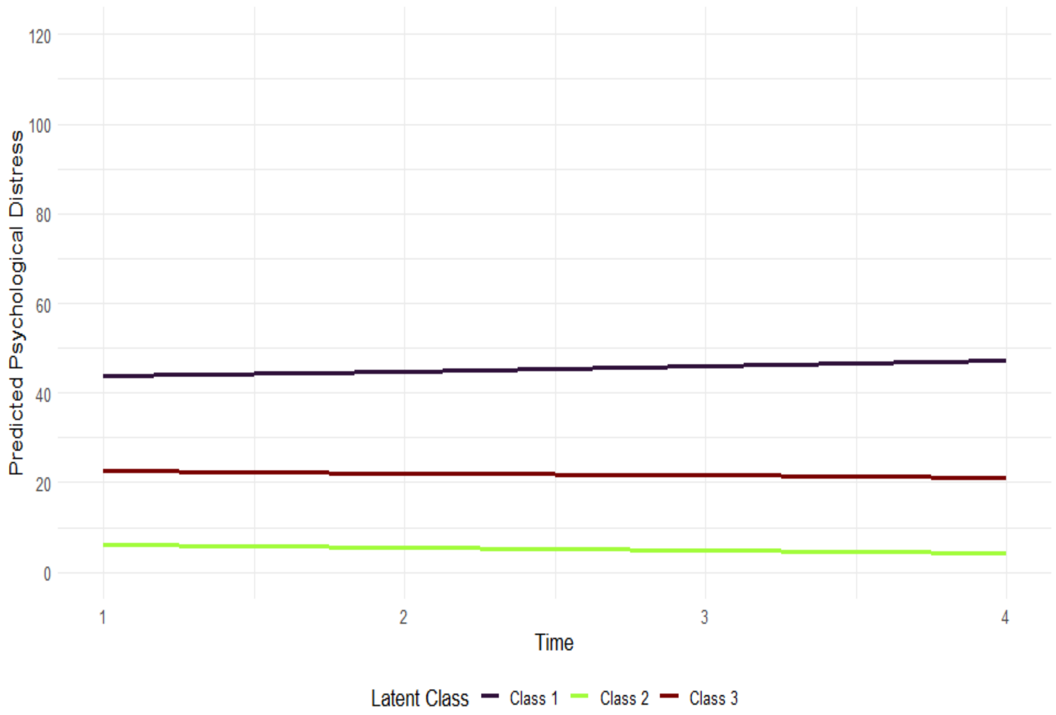

Figure S11. Predicted splines of psychological distress GBTM, linear with no random effects consisting of 3 clusters.

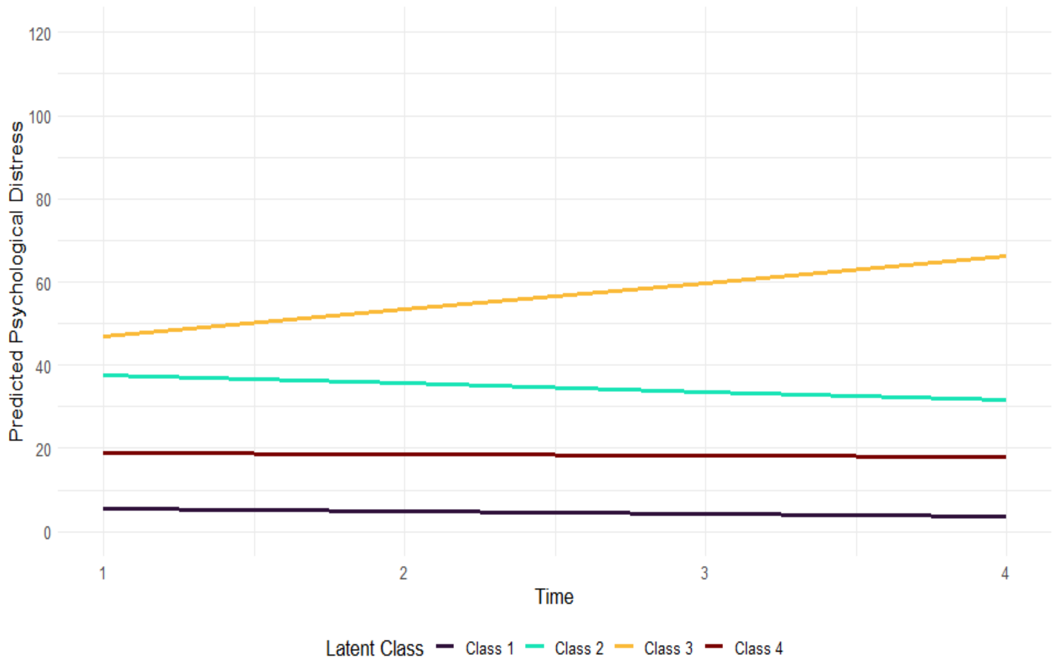

Figure S12. Predicted splines of psychological distress GBTM, linear with no random effects consisting of 4 clusters.

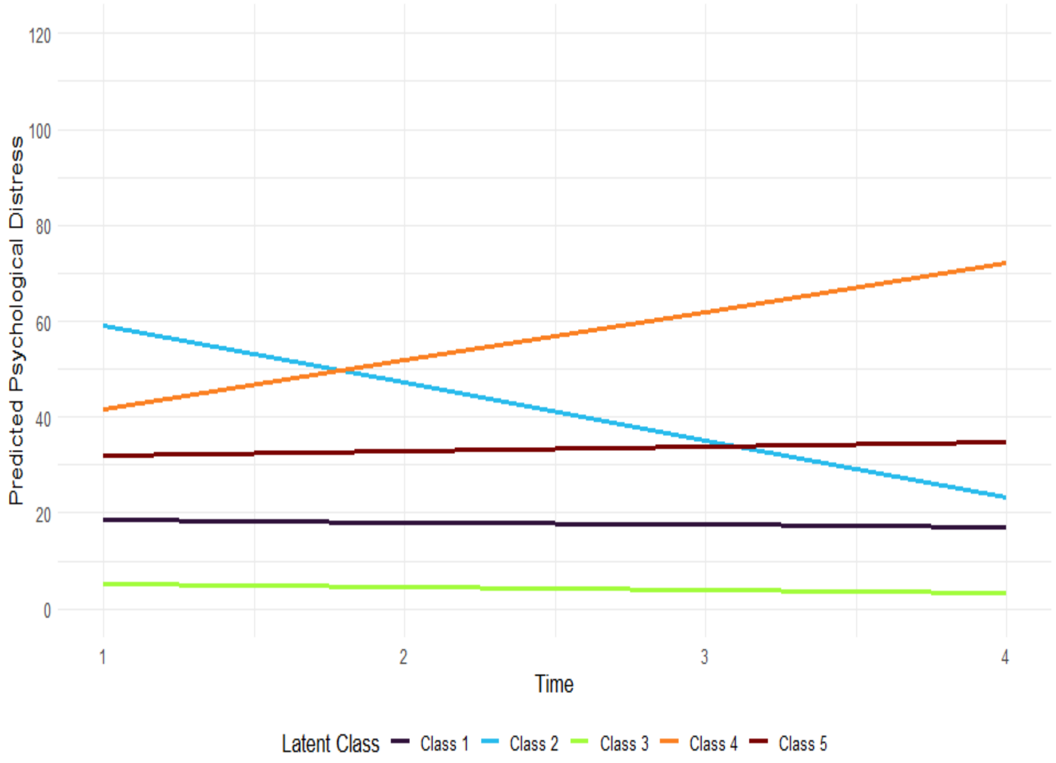

Figure S13. Predicted splines of psychological distress GBTM, linear with no random effects consisting of 5 clusters.

No. clusters	SA BIC	Entropy	Posterior Probabilities	Participants (%)
Linear, no random effects (GBTM)				
1	11368	1.0	NA	100
2	10319	0.83	0.96 / 0.93	67 / 33
3	1072	0.82	0.94 / 0.92 / 0.87	55 / 11 / 34
4	10029	0.75	0.79 / 0.91 / 0.92 / 0.73	20 / 9 / 50 / 21
5	9994	0.74	0.76 / 0.71 / 0.88 / 0.92 / 0.72	10 / 10 / 9 / 49 / 22
6	9986	0.78	0.73 / 0.78 / 0.79 / 0.92 / 0.87 / 0.76	23 / 13 / 2 / 48 / 8 / 6
Linear, random intercept (GMM1)				
1	10171	1.0	NA	100
2	10013	0.57	0.81 / 0.93	47 / 53
3	9949	0.56	0.84 / 0.77 / 0.74	48 / 28 / 24
4	9945	0.56	0.57 / 0.77 / 0.84 / 0.71	21 / 22 / 47 / 10
5	9944	0.58	0.70 / 0.71 / 0.58 / 0.85 / 0.70	5 / 6 / 27 / 45 / 17
6	9955	0.62	0.88 / 0.69 / 0.44 / 0.68 / 0.56 / 0.67	47 / 5 / 7 / 6 / 19 / 16
Linear, random intercept and slope (GMM2)				
1	10167	1.0	NA	100
2	9940	0.65	0.85 / 0.94	46 / 54
3	9931	0.55	0.86 / 0.82 / 0.63	45 / 26 / 29
4	9937	0.62	0.61 / 0.85 / 0.81 / 0.66	22 / 47 / 29 / 2
5	9950	0.58	0.87 / 0.48 / 0.56 / 0.74 / 0.57	47 / 11 / 17 / 15 / 10
6	9947	0.73	0.80 / 0.62 / 0.89 / 1.0 / 0.86 / 0.69	23 / 19 / 46 / 0 / 9 / 3

Table S1. SA BIC, posterior probabilities, and proportions of spinal pain intensity, linear, no polynomial curve. SA BIC Sample-size Adjusted Bayesian Information Criterion.

No. clusters	SA BIC	Entropy	Posterior Probabilities	Participants (%)
Second order polynomial (quadratic), no random effects (GBTM)				
1	11365	1.0	NA	100
2	10314	0.83	0.96 / 0.93	67 / 33
3	10063	0.82	0.87 / 0.94 / 0.92	34 / 55 / 11
4	10017	0.75	0.78 / 0.72 / 0.93 / 0.89	20 / 20 / 51 / 9
5	9989	0.75	0.70 / 0.76 / 0.92 / 0.72 / 0.86	9 / 11 / 50 / 22 / 8
6	10003	0.64	0.70 / 0.86 / NA / 0.74 / 0.71 / 0.76	9 / 8 / 0 / 49 / 23 / 11
Second order polynomial (quadratic), random intercept (GMM1)				
1	10172	1.0	NA	100
2	10085	0.72	0.94 / 0.86	74 / 26
3	10028	0.73	0.73 / 0.81 / 0.92	9 / 22 / 69
4	10042	0.46	NA / 0.68 / 0.58 / 0.77	0 / 11 / 65 / 24
5	10002	0.53	0.70 / 0.69 / NA / 0.54 / 0.85	19 / 16 / 0 / 55 / 10
6	10030	0.32	NA / NA / 0.26 / NA / 0.72 / 0.61	0 / 0 / 60 / 0 / 27 / 13
Second order polynomial (quadratic), random intercept and slope (GMM2)				
1	10176	1.0	NA	100
2	10067	0.74	0.87 / 0.94	23 / 77
3	10082	0.37	0.57 / 0.82 / NA	74 / 26 / 0
4	10096	0.29	NA / 0.40 / 0.79 / NA	0 / 72 / 28 / 0
5			Convergence error	
6			Convergence error	

Table S2. SA BIC, posterior probabilities, and proportions of spinal pain intensity, quadratic curve.

SA BIC Sample-size Adjusted Bayesian Information Criterion.

No. clusters	SA BIC	Entropy	Posterior Probabilities	Participants (%)
Third order polynomial (cubic), no random effects (GBTM)				
1	11368	1.0	NA	100
2	10318	0.83	0.93 / 0.96	33 / 67
3	10072	0.82	0.92 / 0.87 / 0.94	11 / 34 / 55
4	10029	0.75	0.79 / 0.91 / 0.73 / 0.92	20 / 9 / 21 / 50
5	9994	0.74	0.71 / 0.72 / 0.88 / 0.92 / 0.76	10 / 22 / 9 / 49 / 10
6	9969	0.78	0.92 / 0.76 / 0.78 / 0.73 / 0.87 / 0.79	48 / 6 / 13 / 23 / 8 / 2
Third order polynomial (cubic), random intercept (GMM1)				
1	10090	1.0	NA	100
2	10033	0.72	0.85 / 0.94	26 / 74
3	9994	0.72	0.80 / 0.72 / 0.93	21 / 11 / 68
4	9967	0.73	0.72 / 0.86 / 0.72 / 0.92	16 / 10 / 17 / 57
5	9984	0.75	0.72 / 0.92 / 0.78 / 0.82 / 0.71	18 / 57 / 5 / 8 / 12
6	9955	0.58	0.62 / 0.71 / 0.78 / 0.82 / NA / 0.68	55 / 19 / 4 / 8 / 0 / 14
Third order polynomial (cubic), random intercept and slope (GMM2)				
1	10187	1.0	NA	100
2	10075	0.76	0.94 / 0.88	79 / 21
3	10032	0.77	0.82 / 0.92 / 0.82	6 / 76 / 18
4	10049	0.66	0.85 / 0.82 / NA / 0.81	76 / 6 / 0 / 18
5	9927	0.77	0.77 / 0.77 / 0.93 / 0.91 / 0.81	10 / 22 / 4 / 53 / 11
6	9996	0.60	NA / 0.80 / 0.68 / 0.74 / 0.80 / 0.69	0 / 8 / 55 / 15 / 4 / 18

Table S3. SA BIC, posterior probabilities, and proportions of spinal pain intensity, cubic curve.
SA BIC Sample-size Adjusted Bayesian Information Criterion.

No. clusters	SA BIC	Entropy	Posterior Probabilities	Participants (%)
Linear, no random effects (GBTM)				
1	21768	1.0	NA	100
2	20590	0.91	0.94 / 0.83	17 / 83
3	20210	0.87	0.96 / 0.88 / 0.94	67 / 27 / 6
4	20011	0.87	0.95 / 0.87 / 0.96 / 0.90	2 / 26 / 62 / 10
5	19916	0.88	0.87 / 0.95 / 0.89 / 0.93 / 0.93	26 / 61 / 9 / 2 / 2
6	19869	0.85	0.93 / 0.77 / 0.95 / 0.87 / 0.95 / 0.81	2 / 20 / 61 / 9 / 2 / 6
Linear, random intercept (GMM1)				
1	20391	1.0	NA	100
2	20199	0.88	0.89 / 0.98	12 / 88
3	20010	0.92	0.88 / 0.86 / 0.98	4 / 6 / 90
4	19918	0.89	0.88 / 0.97 / 0.96 / 0.81	4 / 2 / 79 / 15
5	19929	0.78	0.89 / 0.88 / 0.97 / 0.81 / NA	78 / 4 / 2 / 16 / 0
6	19884	0.83	0.81 / 0.88 / 0.92 / 0.91 / 0.95 / NA	16 / 4 / 77 / 2 / 1 / 0
Linear, random intercept and slope (GMM2)				
1	20365	1.0	NA	100
2	20170	0.92	0.92 / 0.98	6 / 94
3	20008	0.89	0.96 / 0.90 / 0.98	80 / 18 / 2
4	20018	0.66	NA / 0.98 / 0.81 / 0.89	0 / 2 / 80 / 18

5	20028	0.71	0.98 / 0.89 / 0.86 / 0.64 / NA	2 / 18 / 80 / 0 / 0
6	19928	0.52	0.98 / NA / 0.86 / 0.56 / 0.82 / NA	18 / 2 / 3 / 0 / 77 / 0

Table S4. SA BIC, posterior probabilities, and proportions of psychological distress, linear, no polynomial curve. SA BIC Sample-size Adjusted Bayesian Information Criterion.

No. clusters	SA BIC	Entropy	Posterior Probabilities	Participants (%)
Second order polynomial (quadratic), no random effects (GBTM)				
1	21771	1.0	NA	100
2	20594	0.91	0.98 / 0.94	83 / 17
3	20217	0.87	0.88 / 0.96 / 0.93	27 / 67 / 6
4	20010	0.88	0.96 / 0.92 / 1.0 / 0.87	62 / 9 / 2 / 27
5	19912	0.88	0.95 / 0.97 / 0.87 / 0.92 / 0.87	62 / 2 / 9 / 2 / 25
6	19864	0.86	0.80 / 0.95 / 0.79 / 0.98 / 0.97 / 0.87	5 / 61 / 21 / 2 / 2 / 9
Second order polynomial (quadratic), random intercept (GMM1)				
1	20394	1.0	NA	100
2	20203	0.88	0.88 / 0.98	12 / 88
3	20019	0.92	0.90 / 0.86 / 0.98	4 / 6 / 90
4	19920	0.89	0.96 / 0.89 / 0.98 / 0.82	80 / 4 / 1 / 15
5	19789	0.92	0.90 / 0.99 / 0.96 / 0.83 / 0.97	4 / 1 / 83 / 11 / 1
6	19717	0.91	0.98 / 0.99 / 0.96 / 0.88 / 0.80 / 0.90	1 / 0 / 80 / 4 / 13 / 2
Second order polynomial (quadratic), random intercept and slope (GMM2)				
1			Converge error	
2			Converge error	
3			Converge error	
4			Converge error	
5			Converge error	
6			Converge error	

Table S5. SA BIC, posterior probabilities, and proportions of psychological distress, quadratic curve. SA BIC Sample-size Adjusted Bayesian Information Criterion.

No. clusters	SA BIC	Entropy	Posterior Probabilities	Participants (%)
Third order polynomial (cubic), no random effects (GBTM)				
1	21773	1.0	NA	100
2	20594	0.91	0.94 / 0.98	17 / 83
3	20218	0.88	0.93 / 0.96 / 0.89	5 / 70 / 25
4	20018	0.88	0.87 / 1.0 / 0.91 / 0.96	26 / 2 / 9 / 63
5	19900	0.89	0.95 / 0.87 / 0.98 / 0.89 / 0.95	61 / 26 / 2 / 9 / 2
6	19818	0.90	0.94 / 0.87 / 0.95 / 0.93 / 1.0 / 0.89	2 / 26 / 61 / 1 / 1 / 9
Third order polynomial (cubic), random intercept (GMM1)				
1	20395	1.0	NA	100
2	20202	0.88	0.98 / 0.90	87 / 13
3	20016	0.92	0.92 / 0.85 / 0.98	4 / 7 / 89
4	19871	0.92	0.91 / 0.91 / 0.97 / 0.85	2 / 3 / 88 / 7
5	19751	0.92	0.84 / 0.97 / 0.85 / 0.91 / 0.92	3 / 87 / 5 / 3 / 2
6	19636	0.92	0.92 / 0.87 / 0.81 / 1.0 / 0.96 / 1.0	2 / 4 / 11 / 0 / 82 / 1
Third order polynomial (cubic), random intercept and slope (GMM2)				
1	20366	1.0	NA	100
2	20113	0.90	0.91 / 0.98	10 / 90

3	19976	0.93	0.84 / 0.98 / 0.93	4 / 90 / 6
4	19876	0.91	0.87 / 0.96 / 0.91 / 1.0	12 / 83 / 4 / 1
5	19789	0.91	0.96 / 0.83 / 1.0 / 0.94 / 0.90	78 / 15 / 1 / 4 / 2
6	19687	0.89	0.90 / 0.85 / 1.0 / 0.95 / 0.83 / 0.92	3 / 5 / 1 / 73 / 15 / 3

Table S6. SA BIC, posterior probabilities, and proportions of psychological distress, cubic curve.
SA BIC Sample-size Adjusted Bayesian Information Criterion.
